# The extracellular matrix protein EMILIN1 silences the RAS-ERK pathway via α4β1 integrin and decreases tumor cell growth

**DOI:** 10.18632/oncotarget.15067

**Published:** 2017-02-03

**Authors:** Teresa Maria Elisa Modica, Orlando Maiorani, Giulio Sartori, Eliana Pivetta, Roberto Doliana, Alessandra Capuano, Alfonso Colombatti, Paola Spessotto

**Affiliations:** ^1^ Department of Translational Research, Experimental Oncology 2 Division, CRO Aviano, National Cancer Institute, Aviano, PN 33081, Italy

**Keywords:** extracellular matrix, gC1q domain, α4β1 integrin, RAS-ERK pathway, proliferation

## Abstract

The extracellular matrix plays a fundamental role in physiological and pathological proliferation. It exerts its function through a signal cascade starting from the integrins that take direct contact with matrix constituents most of which behave as pro-proliferative clues. On the contrary, EMILIN1, a glycoprotein interacting with the α4β1 integrin through its gC1q domain, plays a paradigmatic anti-proliferative role. Here, we demonstrate that the EMILIN1-α4 interaction de-activates the MAPK pathway through HRas. Epithelial cells expressing endogenous α4 integrin and persistently plated on gC1q inhibited pERK1/2 increasing HRasGTP and especially the HRasGTP ubiquitinated form (HRasGTP-Ub). The drug salirasib reversed this effect. In addition, only the gC1q-ligated α4 integrin chain co-immunoprecipitated the ubiquitinated HRas. Only epithelial cells transfected with the wild type form of the α4 integrin chain showed the EMILIN1/α4β1/HRas/pERK1/2 link, whereas cells transfected with a α4 integrin chain carrying a truncated cytoplasmic tail had no effect. In this study we unveiled the pathway activated by the gC1q domain of EMILIN1 through α4β1 integrin engagement and leading to the decrease of proliferation in an epithelial system.

## INTRODUCTION

Integrins are heterodimeric cell membrane receptors which mediate cell interaction with the extracellular matrix (ECM). The integrins sense the composition and the mechanical tension within the pericellular milieu, and transmit this information intracellularly to signalling pathways which regulate cell fate by influencing cellular motility, apoptosis, differentiation as well as cell proliferation [1]. Upon engagement integrins rapidly activate signaling pathways mediated by their cytoplasmic domains and proteins with enzymatic activities like RasGTPases are recruited to the sites of cell adhesion to ECM. Integrin engagement by ECM, besides activating the RAS/ERK pathway, also can lead to mobilization of intracellular Ca^2+^ and influx from the extracellular space [2, 3]. While the early events upon integrin engagement have been deeply investigated, less known are the downstream consequences during persistent cell adhesion. In most cell types the ERK pathway which is activated by RAS is one of the key pathways required to trigger cell proliferation. In order to exert its function RAS is farnesylated and then it associates with the plasma membranes [4]. RAS has the inherent ability to undergo conformational changes and functions as a molecular switch that catalyzes the hydrolysis of GTP returning it to the inactive GDP-bound state [5]. Among RAS inhibitors shown to be promising in clinical trials [6], salirasib, that is a farnesylthiosalicylic acid specific H-RAS inhibitor, dislodges the RAS isoforms from their membrane-anchoring sites, thereby preventing the activation of the RAS signaling cascade [7, 8].

EMILIN1 (Elastic Microfibril Interface Located ProteIN) is a homotrimeric ECM multidomain glycoprotein associated with elastic fibers [9] and particularly abundant in skin. EMILIN1 is characterized by a region homologous to the globular domain of C1q (gC1q domain) at the C-terminal end [10, 11] involved in EMILIN1 oligomerization [12] and in cell adhesion and migration via interaction with the α4β1 integrin [13]. To investigate the role of EMILIN1 *in vivo* we generated KO mice and found that the lack of EMILIN1 results in: i) a mild phenotype of lymphatic capillaries [14] and vessels [15, 16] with increased lymphatic endothelial cell proliferation and ii) a skin hyperproliferative phenotype [17].

Ligand-activated integrins in general positively regulate cell growth [18–20]. In contrast, signals emanating from EMILIN1-ligated α4β1 integrin are anti-proliferative due to the increased levels of PTEN and decrease of pEKR1/2 [17]. However, the pathway(s) through which integrin-bound EMILIN1 exerts its negative regulatory function on cell proliferation remain(s) to be fully clarified. For instance, the role and the link between α4β1 and the small GTPases has not been determined yet.

In the present study, in order to mimic the *in vivo* conditions of cell-ECM interaction, we used a persistent cell adhesion approach to further investigate how the α4β1/EMILIN1 pair controls cell proliferation. We found that this is exerted via downmodulation of the HRas pathway through the HRasGTP ubiquitination.

## RESULTS

### Downstream effects of persistent cell adhesion to gC1q

**Figure 1:**
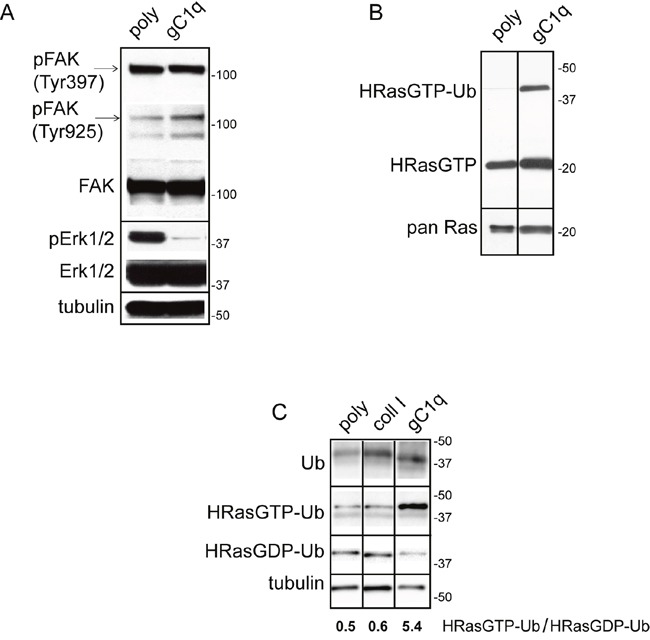
Persistent adhesion to gC1q downmodulates pERK1/2 and upregulates “HRasGTP-Ub” **A**. HaCat cells were lysed 24 hours after plating on polylysine (poly) or gC1q coated plates. The blots were incubated with the indicated antibodies. Tubulin was used as loading control. **B**. Western blot analysis of proteins extracted from HaCat cells 24 hours after plating on polylysine (poly) or gC1q and incubated on a Raf-GST resin. Pan Ras was used to normalize loading. **C**. Western blot analysis of proteins extracted from HaCat cells 24 hours after plating on polylysine (poly), collagen type I or gC1q and incubated on a Raf-GST resin. Tubulin was used as loading control.

Phosphorylation of FAK(Tyr925) generates a binding site for SH2 bearing molecules and triggers a RAS-dependent activation of the MAP kinase pathway [21]. In HaCat keratinocytes persistently adherent to gC1q (i.e. after 24h adhesion) FAK(Tyr925) resulted increased if compared to polylysine attachment, whereas FAK(Tyr397) did not change (Figure 1A). On the contrary, pERK1/2 was dramatically reduced on gC1q at these late times of adhesion. To test if the active form of HRas (HRasGTP) was involved in cellular signals triggered by EMILIN1/gC1q, first a HRasGTP pull-down assay was performed on HaCat cells plated on gC1q or polylysine and analyzed 24h after plating. HRasGTP increased in cells plated on gC1q compared to polylysine (Figure 1B).

In addition to the HRasGTP 21 kDa band, an upper band of about 40 kDa was detected by the anti HRas antibody (Figure 1B). Beside the canonical 21 kDa, RAS proteins display a plethora of faster and slower bands corresponding to the post-translational modified forms or to the mono- (30 kDa) and di-ubiquitinated (40 kDa) forms, respectively [22–25]. In our experiments the intensity of the 40 kDa band, corresponding to the di-ubiquitinated HRasGTP (HRasGTP-Ub) form, was several-fold higher on gC1q compared to polylysine, suggesting that, following α4β1 integrin activation as a consequence of cell adhesion to gC1q, HRasGTP could be ubiquitinated.

### The HRas-ERK1/2 pathway is inactivated via the α4 integrin chain

To determine if the downstream consequences of cell adhesion to gC1q were dependent on a mechanism specifically due to the ligation of the α4β1 integrin, we compared the extent of di-ubiquitinated HRasGTP in cells adherent to gC1q to that detected in cells adherent to collagen type I that uses a different integrin as cellular receptor. As depicted in Figure 1C both the anti ubiquitin antibody as well as the anti HRas antibody recognized proteins whose migration corresponded to the HRasGTP di-ubiquitinated form. HRasGTP-Ub was 10 times less in cells adherent to polylysine as well as to collagen type I compared to gC1q (Figure 1C), suggesting that the α4-dependent signaling was qualitatively/quantitatively distinct and specific. Accordingly, while the ratio of HRasGTP-Ub/HRasGDP-Ub was about 0.5 in cells adherent to either polylysine or collagen type I, this ratio was 10-fold in cells adherent to gC1q, indicating that in α4-dependent cell adhesion there is a specific switch from RasGDP to RasGTP.

### The gC1q ligated α4 integrin chain is physically linked to HRas and controls cell growth

**Figure 2:**
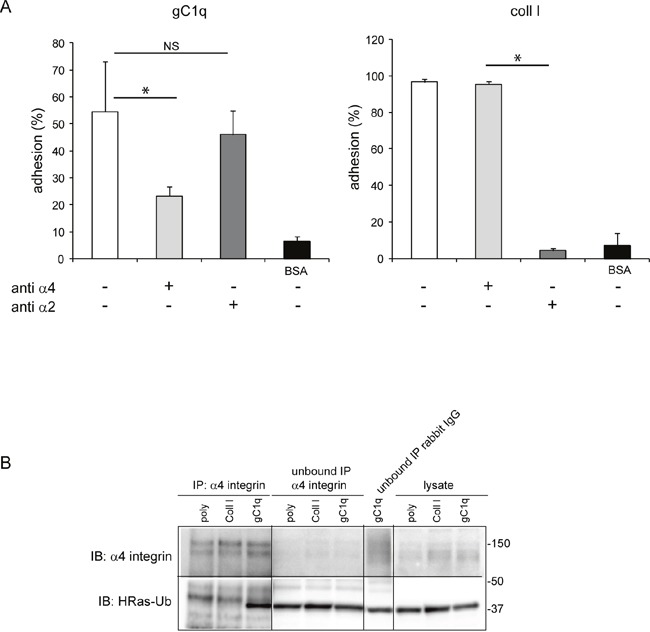
HRas-Ub is co-immunoprecipitated with the α4 integrin chain **A**. Cell adhesion (CAFCA assay) of HaCat cells to gC1q or collagen type I (Coll I) or BSA as negative control. The indicated antibodies were used to block the specific integrin binding. **B**. HaCat cells were lysed 24 hours after plating on polylysine (poly), Coll I or gC1q coated plates. Lysates were immunoprecipitated with the α4 antibody and immunoblotted with anti α4 antibody and anti HRas antibody.

To proof the physiological link between cell adhesion via the α4β1 integrin and HRas we used HaCat cells that endogenously express the α4 integrin chain. We confirmed that the adhesion of HaCat cells to the gC1q or the collagen type I ligands was due to the α4β1 or the α2β1 integrins, respectively, by performing cell adhesion in the presence of the specific integrin blocking antibodies. Thus, the α4β1 mAb greatly reduced cell adhesion to gC1q and the α2β1 mAb fully inhibited cell adhesion to collagen type I (Figure 2A).

Cells were placed on polylysine, collagen type I, or gC1q for 24 hours and at the end of the incubation the cells lysates were immunoprecipitated with the α4 antibody and immunoblotted with anti HRas antibody. As expected, the α4 integrin chain was immunoprecipitated from cells adherent on each ligand; however, the ubiquitinated form of HRas (40 kDa mass) co-immunoprecipitated with the α4 integrin chain only in cells adhered to gC1q (Figure 2B). In fact, a band at the expected molecular mass was detected in the lysate and in the unbound cell lysate when probed with anti HRas antibody. Here, the HRasGTP and HRasGDP ubiquitinated forms cannot be distinguished since the lysates were co-immunoprecipitated and not pulled down for HRasGTP. However, as shown in Figure 1C where the amount of HRasGTP-Ub is much higher than the amount of HRasGDP-Ub, it is very likely that the co-immunoprecipitated HRas-Ub, in cells adhered to gC1q, is primarily due to the HRasGTP membrane-bound form.

**Figure 3:**
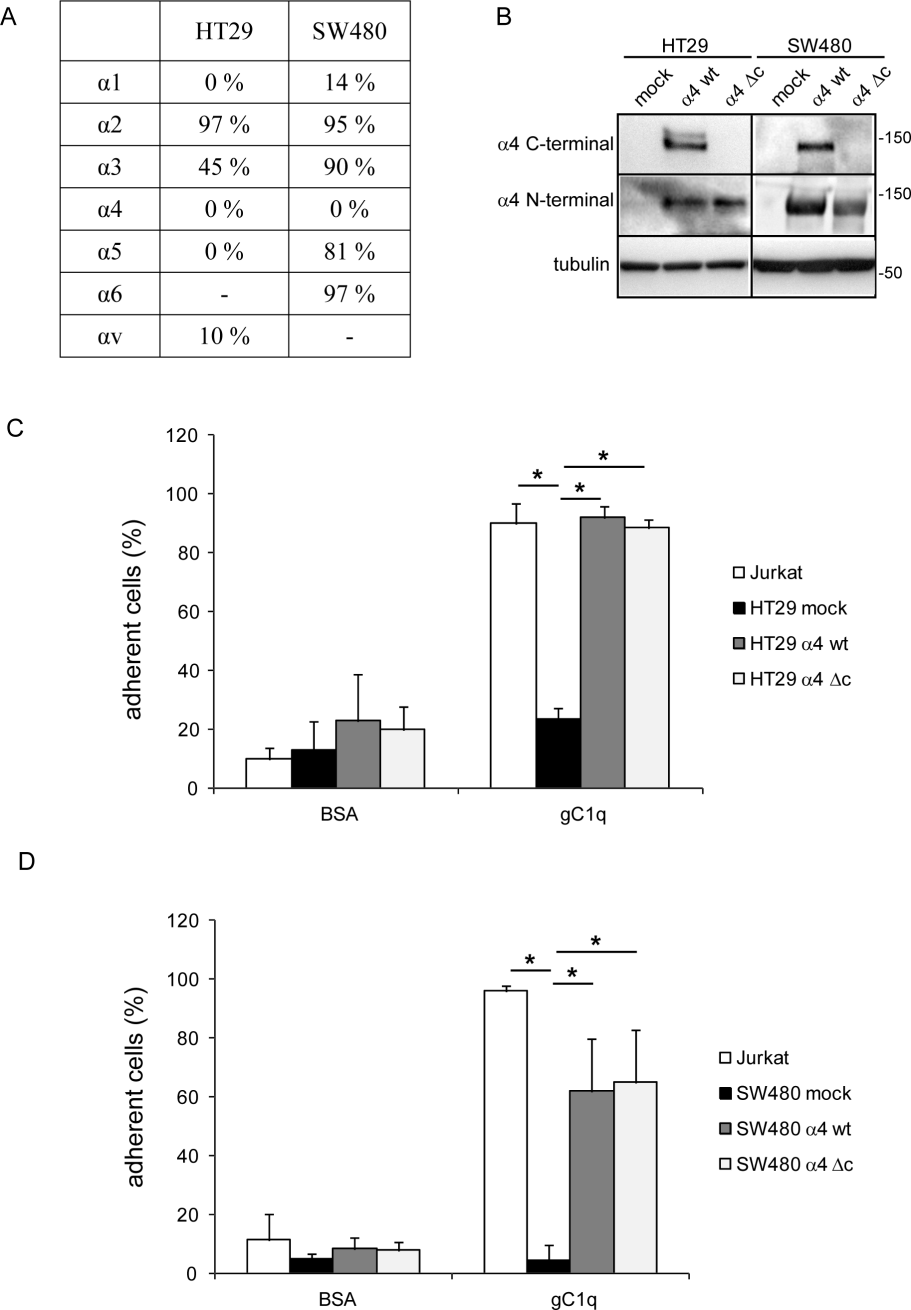
Cells adhere to gC1q through both wt and truncated α4 integrin chain **A**. Endogenous integrin expression panel of HT29 and SW480 cells. **B**. Western blot detection of α4 integrin chain in HT29 and SW480 mock cells, wild type (wt) or truncated (Δc) α4 integrin chain transfected HT29 and SW480 cells. **C**, **D**. HT29 and SW480 cells transfected with the wild type (wt) or truncated (Δc) α4 integrin chain were allowed to adhere to gC1q or BSA as control in CAFCA miniplates. Jurkat cells and mock cells were used as positive and negative controls of adhesion respectively. Data are expressed as the means ± SD of n= 3 independent experiments with n = 6 replicates. *, P < 0.05.

**Figure 4:**
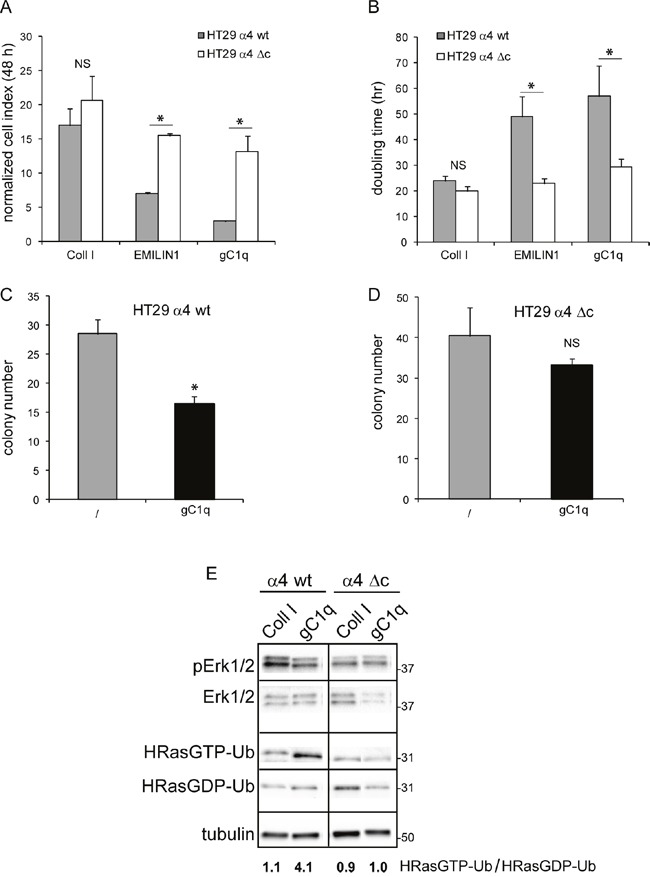
Cells growth is decreased by gC1q Cellular growth of HT29 cells transfected with the wild type (wt) or the deleted (Δc) α4 integrin chain and plated on collagen type I (Coll I), EMILIN1, or gC1q expressed as either normalized cell index **A**. or doubling time **B**. and calculated as the mean ± SD from n = 3 experiments with n = 6 replicates. Cells were allowed to proliferate for 48 hours. Cell index curves were then normalized (normalized cell index) to the point corresponding to 1 hour after plating (maximum cellular adhesion) in order to express proliferation ability independently from attachment to a particular substrate. **C**. HT29 cells transfected with the wild type (wt) or **D**. the deleted (Δc) α4 integrin chain and plated in 0.4% agar in the presence or in the absence of gC1q. **E**. Modulation of HRas-Ub and pERK1/2 in α4 integrin chain transfected HT29 cells. HT29 cells were lysed 24 hours after plating on collagene type I (Coll I) or gC1q coated plates. The blots were incubated with the indicated antibodies (see under Material and Methods). Tubulin was used as loading control. Cell lysates were pulled down on a Raf-GST resin to isolate HRasGTP-Ub.

To demonstrate if this pathway (HRasGTP → HRasGTP-Ub → pERK1/2) was directly linked to the α4 integrin chain, we selected the HT29 and SW480 cell lines that were negative for α4β1 and positive for α2β1 integrin (Figure 3A). Both cell lines were then stably transfected with either the wild type or an α4 integrin chain deleted of the intracytoplasmic sequences (Figure 3B). For the following experiments only cells that resulted at least 95% positive for the expression of the α4 integrin chain were used. Both HT29 and SW480 transfected with either the wild type or the deleted α4 form were able to adhere to gC1q although HT29 cells were more efficient than SW480 cells as the percentage of adhesion to gC1q was 90% and 60%, respectively (Figure 3C and 3D). Thus, for the following experiments we selected HT29 cells. Several clones transfected with either the wild type or the mutant α4 integrin chain were selected and the clones in which the α4 cell surface expression was more stable were further used. We measured the proliferative capacity of the transfected cells grown on collagen type I or EMILIN1/gC1q and found that the proliferation rate (measured as either normalized cell index or doubling time) of the cells transfected with either the wild type or the mutated α4 form was very similar on collagen type I. On the contrary, the proliferation rate was significantly lower on the full length EMILIN1 or gC1q in cells transfected with the wild type α4 integrin chain compared to cells transfected with the deleted form in which the proliferation was not affected at all (Figures 4A and 4B). These results show that the downstream signaling leading to reduced proliferation following α4β1-dependent cell adhesion to the EMILIN1/gC1q ligand depended on a fully functional α4 integrin chain. To link the proliferative and clonogenic capacity to the α4β1 integrin-dependent HRas activity we placed HT29 transfected clones in agar in the presence or in the absence of soluble gC1q. Whereas the colonies of the cells transfected with the wild type α4 integrin chain were significantly less in the presence of gC1q (Figure 4C), the colonies of HT29 transfected with the mutant α4 integrin grew to the same number under all the experimental conditions (Figure 4D).

Moreover, the analysis of extracts from HT29 cells showed that the ratio of HRasGTP-Ub/HRasGDP-Ub in cells stably transfected with the wild type α4 integrin chain and adherent on collagen type I for 24 h was around 1.0; on the contrary and in analogy with data obtained on HaCat cells (see Figure 1C), this ratio was around 4.0 in cells adherent on gC1q (Figure 4E). Our explanation is that after its activation HRas-GTP was directed towards ubiquitination. The ratio of HRasGTP-Ub/HRasGDP-Ub in cells transfected with the deleted α4 integrin chain and adherent on gC1q decreased accordingly compared to cells transfected with the wild type α4 integrin chain (Figure 4E), confirming that this pathway depended on the presence of a fully functional α4 integrin chain.

**Figure 5:**
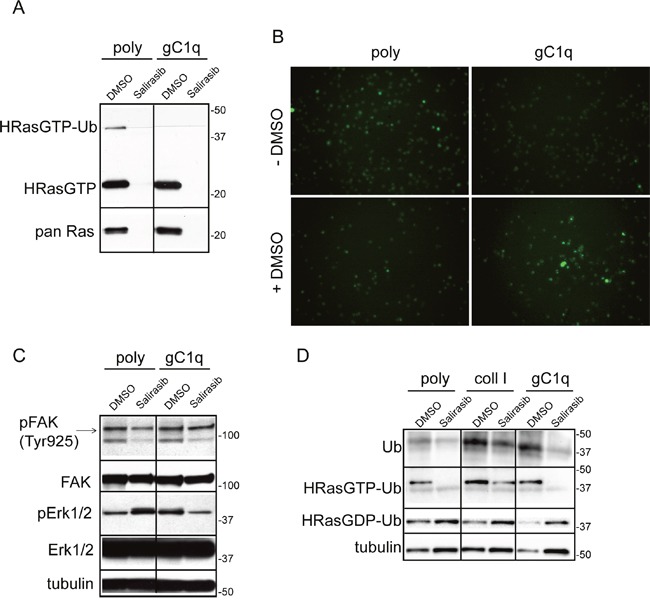
Salirasib/DMSO treatment had opposite effects on cells persistently adherent to polylysine or gC1q **A**. Western blot analysis of proteins extracted from HaCat cells 24 hours after plating on polylysine (poly) or gC1q and incubated on a Raf-GST resin. PAN Ras was used to normalize loading. During the plating the cells were treated (for 24 hours) with DMSO (as vehicle) or salirasib. **B**. Cytoplasmic Ca^2+^ levels decrease in HaCat cells persistently adherent to gC1q. HaCat cells were allowed to adhere to gC1q or polylysine for 24 hours in the absence or in the presence of DMSO; the day after cells were loaded with Fluo-4 AM and the fluorescence intensity was measured by a time lapse instrument. Original magnification 20X. **C**. HaCat cells were lysed 24 hours after plating on polylysine (poly) or gC1q coated plates and treated with DMSO or salirasib. The blots were incubated with the indicated antibodies (see under Material and Methods). **D**. Western blot analysis of proteins extracted from HaCat cells 24 hours after plating on poly, coll I or gC1q, treated with DMSO or salirasib and incubated on a Raf-GST resin. Note that salirasib/DMSO effect on di-ubiquitinated HRasGTP is dependent on the coating. Tubulin was used as loading control.

To prevent HRas pathway activation we interfered with the membrane anchoring of HRas after its farnesylation by treating cells with salirasib. Accordingly, after salirasib treatment both HRasGTP as well as the HRasGTP-Ub forms were detected neither in cell adherent to gC1q nor to polylysine (Figure 5A).

Integrin engagement by ECM constituents leads to changes of Ca^2+^ levels by internal release and external influx that are responsible for controlling a plethora of cellular processes [2, 26–28]. Thus, FLUO-4 AM was loaded in HaCat cells to determine if there was any variation of cytosolic Ca^2+^ levels during the modulation of the HRasGTP/GDP cycling. Cells persistently plated on polylysine exhibited a higher cytoplasmic amount of Ca^2+^ respect to cells plated on gC1q (Figure 5B). When DMSO, that is used as vehicle of salirasib, was added to the cells as control, the cytoplasmic Ca^2+^ levels were changed, i.e. very low in cells persistently adherent to polylysine and, on the contrary, high in cells adherent to gC1q (Figure 5B). DMSO increased the 40 kDa band, likely the di-ubiquitinated HRasGTP (HRasGTP-Ub), in cells adherent to polylysine (see Figure 1B), whereas this band disappeared in cells adherent to gC1q (Figure 5A). Concomitantly, DMSO upregulated pERK1/2 in cells plated on gC1q, respect to polylysine (Figure 5C). On the other hand, salirasib reverted the downstream effects on both types of coatings respect to the DMSO alone. Of note the finding that the levels of pERK1/2 showed an inverse correlation with the 40 kDa band intensity.

We then investigated if the inhibition of HRas membrane anchoring and activation by salirasib affected downstream signaling related to cell proliferation. While there was no significant effect on pFAK levels (Figure 5C), the phosphorylation of ERK1/2 was increased on polylysine when HRasGTP was inibited by salirasib; in contrast, this treatment decreased pERK1/2 levels in cells adherent to gC1q respect to control (Figure 5C). The treatment with salirasib greatly decreased ubiquitination of HRasGTP and increased cytosolic HRas (HRasGDP) on cell plated on either polylysine or gC1q (Figure 5D).

**Figure 6:**
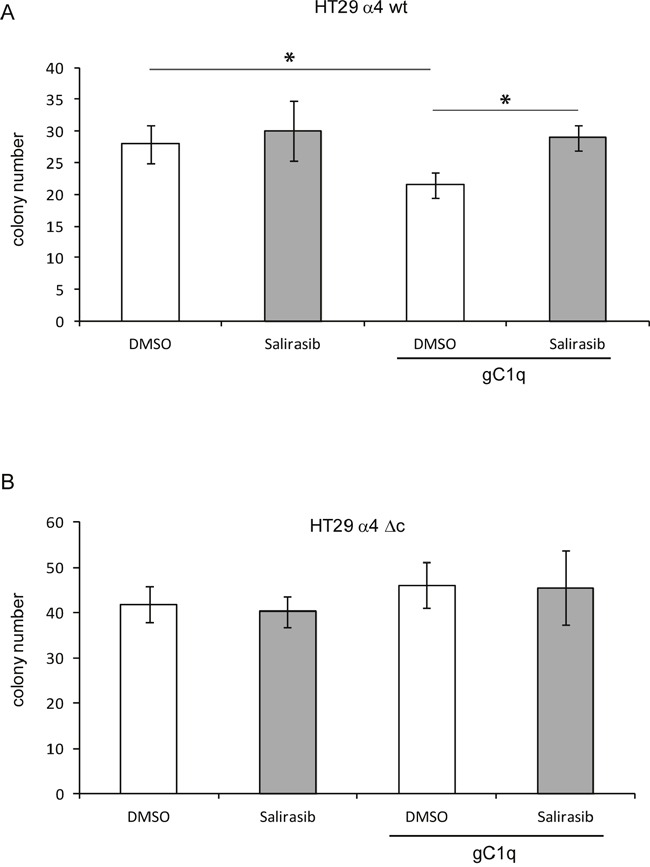
Cells growth in the presence of gC1q is rescued by salirasib **A**. HT29 cells transfected with the wild type (wt) or **B**. the deleted (Δc) α4 integrin chain and plated in 0.4% agar in the presence or in the absence of gC1q and treated with salirasib or DMSO as control. *, P < 0.05.

The number of colonies grown in agar of HT29 cells transfected with the wild type α4 integrin chain was significantly reduced only when plated in the presence of gC1q (Figure 6). The rescue effect by salirasib was not observed under all other conditions, i.e. salirasib and cells transfected with the truncated α4 integrin chain, DMSO with both types of cells. This demonstrated that, by knocking down HRasGTP and thus HRasGTP-Ub, the signalling produced by α4 integrin engagement loses its antiproliferative cues.

## DISCUSSION

**Figure 7:**
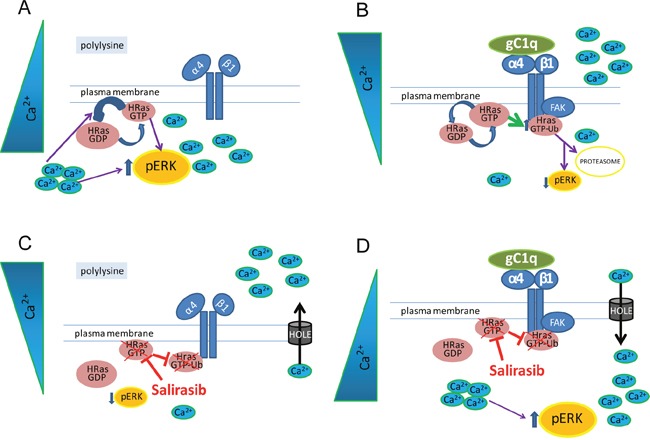
A schematic view of the HRas involvement in gC1q-α4β1 integrin mediated pathway **A**. In cells plated on polylysine the α4β1 inactive integrin is not able to induce HRas activation. High basal level of intracellular Ca^2+^ is known to modulate the activity of RAS and to activate ERK1/2. **B**. Activation of α4β1 integrin, mediated by gC1q, through FAK induces HRasGTP increase as well as its ubiquitination (HRasGTP-Ub). HRasGTP switching off along with low basal level of intracellular Ca^2+^ is able to reduce ERK1/2 phosphorylation and proliferation. **C**, **D**. Salirasib dislodges HRas from plasma membrane preventing its activation and ubiquitination. On the other hand, low levels of cytoplasmic Ca^2+^, in cells plated on polylysine, lead to low ERK1/2 phosphorylation; on the contrary, high levels of cytoplasmic Ca^2+^, in cells plated on gC1q rescue the level of pERK1/2.

We demonstrated here that epithelial cells expressing the α4β1 integrin and persistently engaged *in vitro* with the ECM glycoprotein EMILIN1/gC1q promoted an integrin-dependent downregulation of HRas signaling. This in turn led to the downregulation of pERK1/2 proliferation signals (see Figure 7 for a schematic representation). The persistent cell adhesion *in vitro* is reminiscent of the *in vivo* condition in which cells are in continuous relationship with the constituents of the ECM. The present findings further explain the peculiar role played by EMILIN1 in the maintenance of cell growth homeostasis in the skin [17]. The EMILIN1/α4β1/HRas/pERK1/2 pathway was specific for the α4β1/EMILIN1 pair since cells persistently adherent to collagen type I via the α2β1 integrin did not show enhanced HRasGTP degradation: consequently, the levels of pERK1/2 were unchanged and the cell proliferation was not affected. Moreover, the finding that the α4 integrin chain was co-immunoprecipitated with HRas-Ub only in cells adherent to gC1q and not to polylysine nor to collagen type I indicated that there was a physical association between the α4 integrin chain and HRas-Ub. The reduction of the downstream signaling by HRas required the full integrity of the α4 integrin chain as HT29 cells transfected with a truncated integrin α4 chain were unable to promote HRasGTP ubiquitination and to affect ERK1/2 phosphorylation and hence cell proliferation.

The fact that in HaCaT keratinocytes persistently adherent on gC1q the salirasib vehicle DMSO increased the low basal Ca^2+^ level leading to reduced HRasGTP-Ub and increased pERK1/2, while the opposite was observed on polylysine, indicates that the effects of DMSO were specifically linked to the α4β1 integrin activation by the gC1q ligand. DMSO can induce transient water pores in cell membranes increasing permeability [29]. Ca^2+^ can easily flow through these pores [30, 31]. It is noteworthy that free cytosolic Ca^2+^ is a positive regulator of Ras GTPase Activating Proteins (GAPs) such as CAPRI and RASAL that are responsible for converting HRasGTP to HRasGDP [32–34]; it reduces the levels of HRasGTP and thus of RAS-dependent ERK phosphorylation [35]. Salirasib, which interferes with membrane achoring of HRas, reverted the downstream effects caused by DMSO on persistently adherent cells.

The salirasib-induced reduction in the amount of HRasGTP was the consequence of its dislodgment with increased cytosolic HRasGDP form. Nevertheless, the activation of the α4β1 integrin after engagement by gC1q and the ubiquitination of HRasGTP downregulated cell proliferation under both 2D and 3D conditions.

In conclusion, the α4β1 integrin engagement by the gC1q triggers a series of events leading to the ubiquitination and degradation of HRasGTP. In turn, the loss of HRasGTP is responsible for pERK1/2 downregulation and low proliferation linking the antiproliferative capability of α4β1 integrin to a proto-oncogene like HRas through the switching off of its activated form.

## MATERIALS AND METHODS

### Cells and extracellular matrix ligands

HaCat (immortalized human keratinocytes) cell line was kindly provided by Dr. Lawrence Banks (ICGEB, Trieste). The human HT29 and SW480 colon cancer cells were obtained from the American Type Culture Collection (Rockville, MD). These cells were maintained in continuous culture in humidified 5% CO_2_ at 37°C using DMEM (Life Technologies, Inc., Gaithersburg, MD) supplemented with glutamine, antibiotics and 10% heat-inactivated FCS (Gibco, Thermo Fisher Scientific, Waltham, MA USA). Rat tail collagen type I (BD, Biosciences, San Jose, CA) and recombinant gC1q prepared as extensively described by us [12] were diluted in PBS at the final concentration of 10 μg/ml and incubated on plastic dishes overnight at 4 C. Polylysine (Sigma-Aldrich, Inc., Milan, Italy) used as control was used at the same concentration.

### Protein extraction and immunoblotting

Cells were starved in DMEM without FCS and then harvested by trypsinization and incubated in complete medium for 1h at 37°C to allow the re-expression of the membrane integrins. Cells were then washed in DMEM without FCS, resuspended in DMEM and plated for 24h on dishes previously coated with collagen type I or gC1q or polylysine as control. In some experiments cells were treated with 28 μM salirasib (Cayman Chemical Company, Cayman) in DMSO. After the desired time, the cells were lysed in 50 mM Tris-HCl, pH 7.5, 100 mM NaCl, 5 mM MgCl_2_, 10% glycerol, 0.1% NP40, 50 mM NaF, 1 mM Na_3_VO_4_, 1 mM EDTA, 50 mM DTT, 25X Complete Protease Inhibitor Cocktail (Roche, Basilea, Switzerland) buffer on ice for 30 minutes. The samples were then centrifuged (13,000 x *g* for 30 minutes at 4°C). The isolated proteins of the supernatant were quantified using the Biorad Protein Assay (Biorad, Hercules, California USA). Total cell lysates were separated on precasted 4-20% gels (Biorad), followed by Western Blot (GE Healthcare, Little Chalfont, UK) incubated for 1h at room temperature in Tris-HCl and Tween 20 (50 mM Tris-HCl, 150 mM NaCl, 0.1% Tween 20, pH 7.5) and 5% *Non-fat Dry Milk* (Biorad). The antibodies used according to the protocols supplied by the manufacturer's were the following: pFAK (Tyr925), ERK, pERK1/2 and α4 C-terminal integrin chain from Cell Signaling (Danvers, MA 01923); pFAK (Tyr397) from Invitrogen (Carlsbad, CA); FAK from BD Biosciences (San Jose, CA); HRas, Ubiquitin and α4 N-terminal integrin chain from Santa Cruz (Dallas, TX); Alpha tubulin from Sigma (Milan, Italy). The proteins were then decorated using horseradish peroxidase-conjugated anti-mouse or anti-rabbit antibodies (GE Healthcare) and autoradigraphed by Immobilion Western Chemoluminecsent HRP Substrate (Millipore, Darmstadt, Germany) or analyzed on Biorad Chemidoc Touch Imaging System and quantified by Quantity One Software densitometry or by Biorad ImageLab.

### Construction of a cytoplasmic tail deleted α4 integrin chain

The cytoplasmic tail-deleted α4 chain mutant R974 was generated by introducing a termination codon after residue R974 into che cDNA by PCR. The full-length α4 integrin chain cloned into the Bluescript vector was used for amplification of the 1-974 region of the α4 integrin chain (α4 forward: 5'ggctcgagccatggcttgggaagcgaggcgccg3’, including a XhoI restriction site followed by a “kozak” consensus sequence at the 5’-end, and α4 reverseTM 5'ggctggcttctttaaaagataatctagagg3’ including a XbaI and XhoI restriction sites) and then subcloned in the pcDNA 3.1 vector (Invitrogen). A clone carrying the insert was sequenced and used in the subsequent experiments. The vectors were transfected into HT29 and SW480 cells using FuGENE HD Transfection Reagent (Promega, Madison, WI) following the manufaturer's protocol. To confirm successful transfection, cell extracts of α4-transfected cells were analyzed by Western Blot analysis using anti N- and anti C-terminal integrin α4 antibodies. To perform experiments with cells uniformly expressing the α4 integrin chain, (more than 95% positive cells), cells were sorted twice by flow cytometry under sterile conditions in a FACS Canto (Becton Dickinson, Franklin Lakes, NJ) using a specific antibody (PE anti-human CD49d; BioLegend, Pacific Heights, San Diego, CA).

### HRas pull down assay

The DH5alpha bacterial strain was transformed with a pGEX-2T bacterial expression vector encoding GST followed by the 2-149 N-terminal residues of human Raf-1 and induced to overexpress the protein by 0.1 mM IPTG treatment overnight at RT under shaking. This plasmid contains the RAS-binding domain (RBD) that binds only to activated Ras-GTP (gift of Channing Der, Addgene plasmid #13338). GST-Sepharose resin (GE Healthcare) was washed in cold PBS and incubated under shaking for 2h at 4°C with total bacterial proteins to allow Raf-RBD conjugation with the resin. The resin was then washed and incubated with cell lysates under shaking for 1h at 4°C. At the end of the incubation, loading buffer (0.25 M Tris-HCl pH 6.8, 10% SDS, 0.5 M dithiothreitol (DTT), 50% glycerol, 0.25% bromophenol blue) was added to each sample before protein denaturation by boiling for 10 minutes. The samples were then loaded onto 4-20% gels followed by Western Blot analysis.

### Soft agar colony formation assay

For the bottom layer 2 ml of 0.6% agar (Sigma) dissolved in PBS and diluted in DMEM with or without 10 μg/ml gC1q were allowed to polymerize on 6 well plates. For the top layer 2 ml 0.4% agar dissolved in PBS and diluted in DMEM with or without 10 μg/ml gC1q were allowed to polymerize. 28 μM salirasib in DMSO or DMSO used as negative control was added to both agar solutions before polimerization. Before mixing with the top agar layer, 5.0 × 10^4^ HT29 cells transfected with the wild type or the deleted mutant α4 integrin chain were resuspended in DMEM containing 10% FCS and mixed with the top agar solution. Cells were allowed to grow for 12 days.

### Proliferation assays

To quantitatively monitor cell behavior in real time, we adopted the xCELLigence technology provided by the Real-Time Cell Analyzer dual plate instrument (Roche) [36]. The cell index, an arbitrary measurement and a reflection of overall cell number, attachment quality, and cell morphology can change as a function of time. For proliferation assays, the E-Plates 96 (Roche) were coated with EMILIN1, gC1q or collagen type I (20 μg/ml) (4°C, overnight). Cells were then seeded at 5 × 10^3^ cells/well in 10% FCS-containing medium. Cells were monitored every hour for 48 hours. Data analysis was performed using Real-Time Cell Analyzer software (version 1.2) supplied with the instrument. Experiments were performed in triplicate.

### Determination of the cytoplasmic Ca^2+^ levels

HaCat cells were starved in DMEM without FCS and then harvested by trypsinization and incubated in complete medium for 1 hour at 37°C. Cells were then washed in DMEM without FCS, resuspended in DMEM and plated for 24 hours on a 96 well plate previously coated with gC1q or polylysine in the presence or in the absence of DMSO. The following day, the cells were loaded with 5 mM Fluo-4 AM (Invitrogen) in DMSO according to the manufacturer's instructions. Fluorescence intensity of the time lapse images was acquired every 45 min during a period of 6 hours using the AF6000 LX workstation Leica.

### Cell adhesion assay

The quantitative cell adhesion assay used in this study is based on centrifugation and has been extensively described [37]. Cells were labeled with the vital fluorochrome calcein AM (Invitrogen) for 15 min at 37°C and then aliquoted into the bottom CAFCA (Centrifugal Assay for Fluorescence-based Cell Adhesion) miniplates at a density of 2.0 to 5.0 × 10^5^ cells/ml. CAFCA miniplates were centrifuged at 1,000 rpm for 5 min at 37°C to synchronize the contact of the cells with the substrate. The miniplates were then incubated for 20 min at 37°C and subsequently centrifuged at 500 rpm. The relative number of cells bound to the substrate (i.e. remaining in the wells of the bottom miniplates) was estimated by top/bottom fluorescence detection in a computer-interfaced InfiniteM1000 PRO miniplate reader (TECAN Group LTD, Mannedorf, CH).

### Statistical methods

Plotted values are shown as means ± standard deviation. Statistical significance of the results was determined by using the two-tailed unpaired Student's t test to determine whether two datasets were significantly different. A value of P < 0.05 was considered significant.

## Abbreviations

ECM: extracellular matrix
